# Payment and Coverage Parity for Virtual Care and In-Person Care: How Do We Get There?

**DOI:** 10.1089/tmr.2023.0014

**Published:** 2023-05-18

**Authors:** Nandita Khera, Meghan Knoedler, Sarah K. Meier, Sarvam TerKonda, Ryan D. Williams, Christopher M. Wittich, Jordan D. Coffey, Bart M. Demaerschalk

**Affiliations:** ^1^ Department of Medicine, Mayo Clinic, Phoenix, Arizona, USA.; ^2^ Revenue Strategy Services, Mayo Clinic, Rochester, Minnesota, USA.; ^3^ Communications Department, Robert D. and Patricia E. Kern Center for the Science of Health Care Delivery, Mayo Clinic, Rochester, Minnesota, USA.; ^4^ Division of Plastic Surgery, Mayo Clinic, Jacksonville, Florida, USA.; ^5^ Federation of State Medical Boards, Euless, Texas, USA.; ^6^ Center for Digital Health, Mayo Clinic, Rochester, Minnesota, USA.; ^7^ Division of General Internal Medicine, Mayo Clinic, Rochester, Minnesota, USA.; ^8^ Department of Neurology, Mayo Clinic College of Medicine and Science, Phoenix, Arizona, USA.

**Keywords:** virtual care, digital health, telehealth, telemedicine, coverage parity, reimbursement parity

## Abstract

**Background:**

A steep increase in the use of delivery of virtual care occurred during the COVID-19 public health emergency (PHE) because of easing up of payment and coverage restrictions. With the end of PHE, there is uncertainty regarding continued coverage and payment parity for the virtual care services.

**Methods:**

On November 8, 2022, The Mass General Brigham held the Third Annual Virtual Care Symposium: Demystifying Clinical Appropriateness in Virtual Care and What's Ahead for Pay Parity.

**Results:**

In one of the panels, experts from Mayo Clinic led by Dr. Bart Demaerschalk discussed key issues related to “Payment and Coverage Parity for Virtual Care and In-Person Care: How Do We Get There?” The discussions centered around current policies around payment and coverage parity for virtual care, including state licensure laws for virtual care delivery and the current evidence base regarding outcomes, costs, and resource utilization associated with virtual care. The panel discussion ended with highlighting next steps targeting policymakers, payers, and industry groups to help strengthen the case for parity.

**Conclusions:**

To ensure the continued viability of virtual care delivery, legislators and insurers must address the coverage and payment parity between telehealth and in-person visits. This will require a renewed focus on research on clinical appropriateness, parity, equity and access, and economics of virtual care.

## Introduction

Virtual care is the deployment and use of technologies to provide home or community-based and office-based care. As a care delivery strategy, virtual care holds huge potential to overcome certain limitations of our current health care system with its focus on fee-for-service environment. Considerable growth was seen in virtual care delivery as the system adjusted to pandemic circumstances and the presence of telehealth flexibilities made available during the public health emergency (PHE) declared by the federal government.^1,2–5^

The subject of virtual care parity is not a new concept. In the wake of the COVID-19 pandemic, virtual care use surged because Medicare and private payers eased payment and coverage restrictions. Many states passed legislation that required insurance plans to include virtual care services, but the extent of coverage and payment parity varied. Although currently there is bipartisan support from lawmakers for the continued use of virtual care following the pandemic, enduring implementation remains a challenge.

The current health care system is not optimally set up to support, compensate, or incentivize virtual care, in all its forms. Despite a strong showcase for the delivery of care through virtual means during the COVID-19 PHE, the regulatory landscape is complex with many unanswered questions: Should virtual care services be covered to the same extent as in-person services and should coverage be provided for additional virtual care services such as remote patient monitoring? In addition, there are significant state-by-state variations as commercial payers, providers, and patients chart their virtual care strategies following a paradigm shift in how care is delivered.

In this review, we highlight current policies around payment and coverage parity for hybrid virtual care and in-person care, state licensure laws for virtual care delivery and challenges for the future as PHE comes to an end. We also review the current evidence base regarding outcomes, costs, and resource utilization associated with virtual care that will likely influence coverage and payment parity. Finally, we reflect on the next steps in the context of results from a survey conducted to identify future research that would provide optimal information to strengthen the need for payment and coverage parity.

## Key Telehealth Policies During the PHE

In the wake of the COVID-19 pandemic, telehealth use surged because Medicare and private payers eased payment restrictions. Importantly, the PHE changed the national landscape of payment parity, coverage parity, and licensure, impacting the overall adoption of telehealth technologies. Beyond the PHE declaration, the Centers for Medicare and Medicaid Services (CMS) acted to establish numerous flexibilities and waivers to provide delivery system stakeholders with the capability to adjust to best meet needs in the context of the COVID-19 pandemic.^6^

There were several telehealth related actions during this time, indicating significant CMS activity to expand coverage and reimbursement for telehealth during the pandemic.^7^ “Action 189” highlights CMS steps to “allow use of audio-only equipment to furnish audio-only telephone E/M, counseling, and educational services” (page 75746). “Payment for Medicare Telehealth Services Under Section 1834(m) of the Act” (listed as “Action 125”) outlines CMS's key effort to add more services in the short-term to CMs' list of telehealth services for Medicare, as well as related steps taken on a short-term basis (Page 75736).

Moving forward, the Consolidated Appropriations Act (CAA), 2023 establishes an extension of many key telehealth policies allowed during the public health emergency through the end of 2024, creating short-term clarity with respect to many key telehealth policies.^8^ In separate PHE wind-down documents provided by CMS, note is made of some telehealth flexibilities that will remain through the end of 2024 due to the CAA, 2023.^9^ However, ultimately the specifics of how long telehealth services will be reimbursable under Medicare after the PHE depends on whether the service is classified in one of the existing Medicare telehealth code categories and which category, as well as potential decisions in future CMS rulemaking.^10^

## Variability in Coverage and Reimbursement Parity Across State Lines

**FIG. 1.**
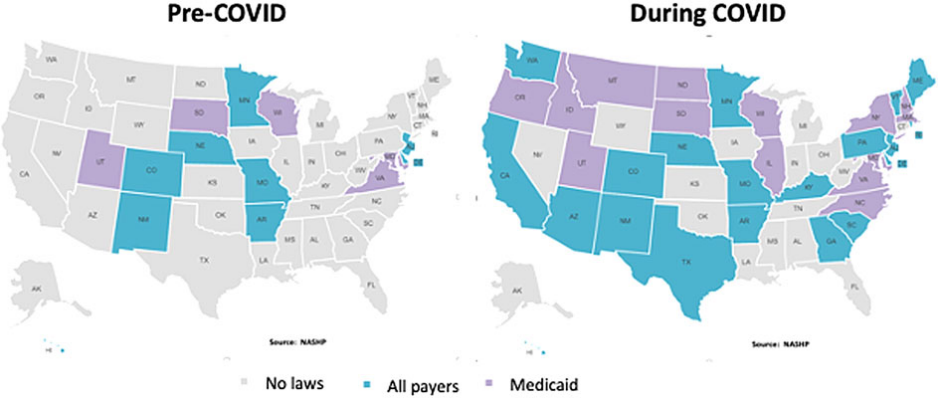
States with laws requiring insurers to implement payment parity.

Before the pandemic, telehealth use was largely limited and restrained by the ambiguous and often changing regulations regarding reimbursement and licensure, especially across state lines. Early in the pandemic, CMS used its waiver authority to establish payment parity for traditional in person visits, video, and audio only telehealth visits during the COVID-19 pandemic. Most commercial payers followed suit. Figure 1 depicts the payment parity laws before the pandemic (pre-COVID) and during COVID.^11^

States in gray had no laws regarding payment parity, and states in purple supported payment parity within Medicaid. Payment parity for all payers is noted in blue. The number of states with payment parity for all payers increased from nine to 21 during the pandemic. Medicaid payment parity increased from 5 to 14 states. As the number of COVID cases subsided, states moved away from the payment parity requirements.

**FIG. 2.**
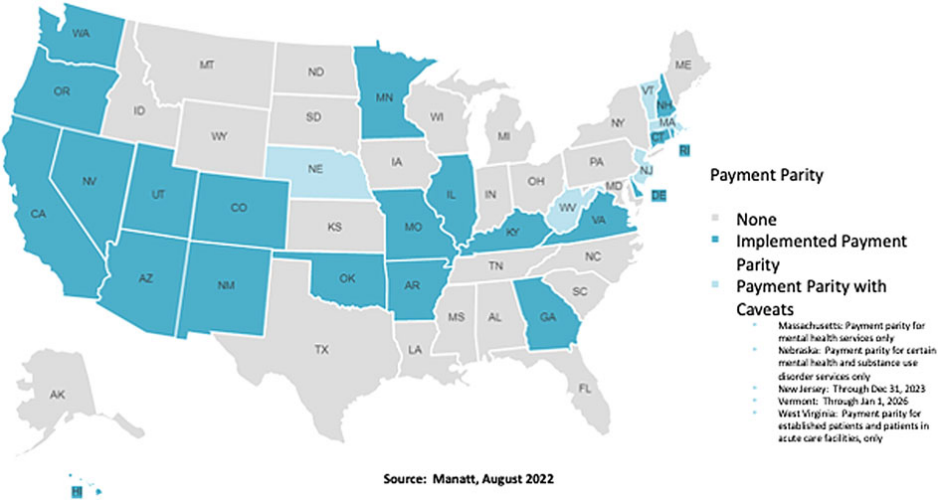
States with laws requiring insurers to implement payment parity as of August 2022.

Figure 2 illustrates the changes in payment parity as of August 2022.^12^ Many states continued with payment parity laws (dark blue, 20 states), whereas other states rescinded the laws (gray states). Some states amended the payment parity laws with specific restrictions such as services or location of patient (light blue, 5 states).

**FIG. 3.**
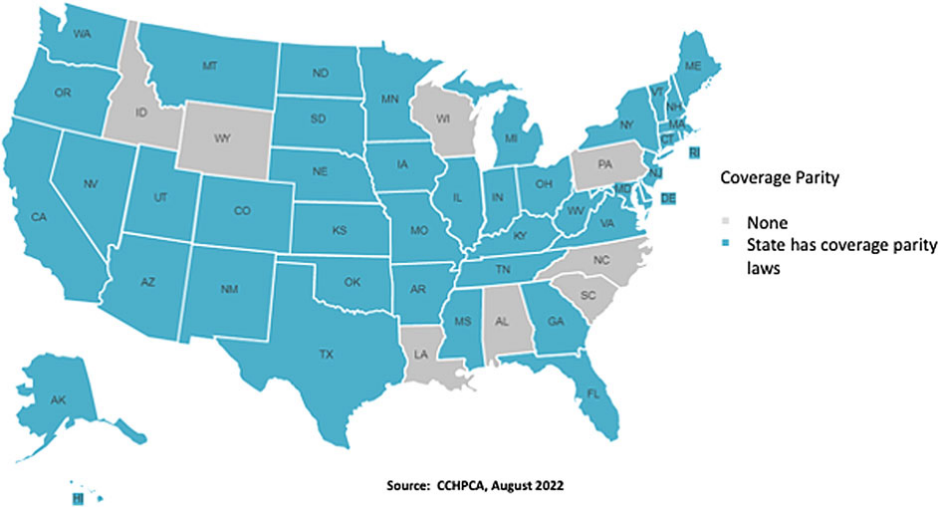
States with laws requiring coverage parity as of August 2022.

Another significant challenge for the adoption of telehealth is coverage parity. Similar to payment parity, there is a lack of consistency in how telehealth services are covered by insurance plans. Some plans offer no coverage, other plans detail specific limitations on services, and others have coverage parity for all services. Figure 3 illustrates states with coverage parity laws as of August 2022.^13^ Forty-two states (blue states) have coverage parity laws, with eight states having no laws regarding parity (gray states).

There may be some variation by specialty in each of the states such as some states (e.g., Massachusetts) require payment parity for behavioral health, but not other services, such as primary care. Providing a comparative analysis of state-by-state approaches and the merits of these provisions is beyond the scope of this article. Although federal and state governments have taken steps to promote both payment and coverage parity, the significant variations in payment and coverage may discourage providers from offering telehealth services.

Before the pandemic, telehealth was also challenged by the requirements for licensure across state lines. These regulations required the health care provider to hold state medical licenses in the state where the patient is located, and some states also require licensure in the state where the clinician is located. To improve access to health care services and facilitate the use of telehealth, many states implemented temporary changes to their licensure requirements during the pandemic.

**FIG. 4.**
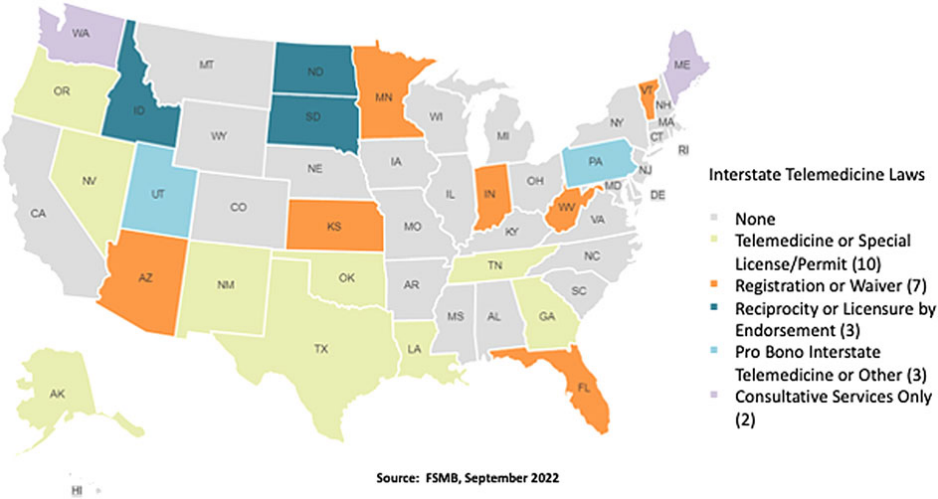
States with permanent interstate telemedicine laws as of September 2022.

These changes included emergency measures temporarily waiving licensure requirements, expediting the temporary licensure process or the creation of special telehealth registration or licensure. Figure 4 illustrates the states with permanent telemedicine laws as of September 2022.^14^ Interestingly, over 50% of states had no permanent interstate telemedicine laws.

However, 10 states implemented a special telemedicine license or special permit (light green states), and 7 states had a registration process or implemented a waiver for telemedicine (orange states). To ease the burden of the licensure process for providers, the Interstate Medical Licensure Compact (IMLC, established in 2014) provides an expedited pathway to licensure while preserving the state's rights to licensure issuance, enforcement, and revenues.

The IMLC does not create a single multi-state license or licensure reciprocity (as exists with a driver's licenses in the US) but simply a common application for some administrative simplification. In 2015, 11 states were participating in the compact. As of August 2022, 37 states, District of Columbia and the US territory, Guam were members of the IMLC.^14,15^

It should be noted that shifting the reimbursement paradigm for virtual care will include a wide range of health care providers beyond the physicians such as nurses, physical therapists, psychologists, occupational therapists, counselors, etc., who may have similar licensure portability compact structures. The precise legal pathways are unique, though still analogous to the example of the IMLC's interplay with local parity laws. State and federal legislation in the use of telehealth laws served as a hindrance to fully realize the benefits of telehealth.

Billing for telehealth services is subject to significant variability in terms of both coverage and reimbursement parity. Most state jurisdictions (44, including Washington, D.C.) have a statue within their state codes governing and defining telehealth. Of these 44, the majority of those include within that definition some degree of coverage parity, requiring a regulated insurer to cover a virtual service if the insurer would have covered the same service when performed in person. (It is important to note that even with this critical mass move coverage parity, the impact of that will shift depending on the text of the state statute, and thus mileage varies).^16^

Even less ubiquitous across 50 states is a codified notion of reimbursement parity—whether the amount paid for the service is commensurate with the in-person equivalent service. Approximately half of the states have codified payment into their state law regulating commercial insurers. States have different approaches and requirements around this issue, with some of them leaving more discretion to the contracting parties to agree on a rate that may distinguish between the modality of the service.

While this approach gives more flexibility to a given payer and provider to have different rates for the same service, it may shift local providers' interest in providing virtual services and thereby potentially create disparate care experiences for patients throughout the state.

## Factors in Consideration for Coverage and Reimbursement Parity

The current fee-for-service system and the underlying infrastructure and regulations are vastly limiting the enormity of transformation potential of virtual care. The interwoven complexities between licensing and credentialing; federal, state, and local regulations; payment and coverage parity, the list of service modalities, the vast number of waivers, and PHE exemptions make it difficult to leverage the advancement of technology and virtual care in a sustainable fashion. Table 1 outlines the challenges and potential solutions for improving payment parity for virtual care. The following factors need to be considered when making a plea for coverage and reimbursement parity.

**Table 1.** Challenges and Potential Solutions for Payment Parity for Virtual Care

Challenges	Potential solutions
Variations in virtual care coverage based on Geography Technology type Setting of patient or provider Insurance, public and private	Innovative payment or service delivery models Parity laws Expanded coverage
Variations in state physician licensure requirements for providing telehealth	Interstate medical licensure compact
Concern about poor quality of care and/or high technology costs associated with virtual care	Rigorous clinical and cost effectiveness studies Increase broadband availability Regulatory requirements Quality benchmarks for virtual care Investment in data safety protocols

(A) Need for Alternative Payment Models (APM)
We require an innovative approach outside the framework of fee-for service system to develop value-based models focused on cost and quality of virtual care utilization to leverage the technological advancements. Expansion of APMs such as bundled payment and Accountable Care organizations has been shown to improve the adoption of virtual care services while decreasing the risk of overuse andfraud.^17^
Even before the pandemic, organizations such as Veterans Health Administration were using non fee-for service models to deliver mental health services using video conferencing.^18^ Creation of an APM for a model of virtual care delivery would be essential to have Medicare billing codes, allowing clinicians to bill directly for associated services and paving the way for broad-scale adoption of the virtual care in the United States.
(B) Resource utilization and costs associated with virtual care
This remains the subject of ongoing research, but existing studies demonstrate reduced length of stay and lower or comparable rates of readmissions, emergency department visits, and skilled nursing facility admissions with certain virtual care models such as hospital at home and remote patient monitoring.^19–21^ A recent cross-sectional study indicated that incorporation of virtual care into the ambulatory care practice was not associated with increased overall visit volumes or additive costs.^22^
There is scant literature on the benefit on patient level costs with telemedicine through avoidance of travel costs and missed work in certain populations.^23^ A more comprehensive evidence base is needed through more rigorous economic assessments from multiple perspectives (patient, clinic and hospital, health system, payer, societal) to understand health care utilization, as the current literature indicates substantial variation in the costs and lack of detailed cost data.^24^
(C) Differences in outcomes between virtual and in person care
It is important to consider the safety and appropriateness of virtual care when thinking about coverage parity. Multiple studies in different area of medicine in varied settings such as outpatient, inpatient, or intensive care unit have shown similar or better clinical outcomes for patients receiving virtual care as compared with in-person care, which strengthens the argument for ensuring coverage and payment parity.^25,26^ A recent systematic review reported similar clinical effectiveness, health care use, patient satisfaction, and quality of life when usual care was replaced or augmented by video-teleconferencing.^27^ A high degree of patient and clinician satisfaction was reported in a recent survey study measuring ratings of overall visit quality and willingness to recommend the visits.^28^

## Next Steps

As we consider everything presented in earlier sections regarding the state of virtual care and digital health services, we recognize the importance of more proactively planning for future conversations with policymakers, payers, and industry groups to make payment parity permanent. Ultimately, we know that objective evidence is vital to informing these conversations with decision makers.

However, we also recognize that we must collect needed data on virtual care use, accessibility, patient satisfaction, quality outcomes, equity, and impact on overall health care spending by conducting rigorous high-quality studies. These data can then be used to shape an informed and thoughtful approach to virtual care payment with consideration of value to all relevant stakeholders.

One of the ways Mayo Clinic Center for Digital Health has approached this challenge is through the development of a 5-year digital health research agenda.^29^ The goal behind this was to ensure that there is a strong understanding of what challenges the field of virtual care may be facing and leaning into those to focus the research efforts and the insights. A modified Delphi approach was developed to understand what top virtual care research themes emerged and required attention.

With that in mind, Mayo Clinic engaged with over 40 digital health leaders across industry, payers, provider organizations, and think tanks to identify top concepts and themes. Those top themes were thematically coded and distributed to an additional 120 American Telemedicine Association members and fellows across the country. Ultimately, this helped to identify a prioritized understanding of where the health care industry should focus some of its research efforts and resources.

Table 2 lists these themes and reflects on what research questions could be asked in that domain. Most highly prioritized themes included clinical appropriateness, parity, equity and access, and economics. Modestly prioritized themes related to licensing, legal and compliance, and patient engagement. Lesser prioritized themes were digital scalability, the potential for fragmentation in the health care system, team dynamics and potential issues of waste, fraud, and abuse (Table 3).

**Table 2.** Top Themes Identified from Stakeholder Feedback with Examples of Topics Covered Within Each Theme

Theme	Description
Clinical appropriateness	What use cases are appropriate for digital health, and what use cases are inappropriate? What needs to be done by a human, and what can be done by a system (automation, Artificial Intelligence/Machine learning, etc.)?
Clinical parity	Are digital health-based approaches inferior, non-inferior, or superior to “traditional” models of care in reference to clinical outcomes? How is digital health impacting the quality of care? Do patients and health care teams trust digital health?
Economics	How does digital health affect the overall cost of care within the health care systems? How do new digital health care models affect efficiency within care pathways? How is digital health impacting health care economic viability (for patients, payers, and providers)? How should digital health be reimbursed? At parity with in-person services? At a greater/lesser level?
Equity/access	Does digital health affect equity and/or disparity in health care? How is digital health impacting the health care divide and patient access? Does digital health drive greater or lesser access to high-value care? How are we ensuring that patients and populations have access to digital tools, high-speed internet and technology to access those tools and that the tools take into account different needs (e.g., multiple languages, appropriate reading levels (6th grade or lower), etc.)? What is the future for partnerships enabled through digital health (e.g., schools, community centers, employers, etc.)?
Fragmentation	Is digital health driving health care toward fragmentation or integration? How do health care organizations ensure integration (with technical systems and clinical processes) rather than a collection of point solutions?
Licensing/legal/compliance	How should licensing for digital health-delivered services be viewed relative to traditional in-person delivered services (e.g., cross-state, multi-state, national, international practices, new vs. follow-up patients, etc.)? How is digital health impacting medical legal risk? What is appropriate legal/safety/regulatory oversight for digital health?
Patient engagement	How does digital health affect patients' willingness to engage in their care (self-efficacy/activation)? Does digital health affect the appropriate use of care? Does digital health impact patients' expectations of care?
Scalability	Is digital health care scalable? How does it impact health care expansion and sustainability? Will health care organizations sustain the pace of change as new technologies become available and patient/provider expectations evolve/increase? What standards are necessary to ensure quality, interoperability, and common understanding/performance expectations among digital health solutions?
Team dynamics	How does digital health positively or negatively impact care team members' ability to collaborate and/or perform at the top of their licensure? How is digital health impacting clinical complexity, cognitive burden, and burnout?
Waste/fraud/abuse	How does digital health affect waste, fraud, and abuse in health care? How does digital health impact the consumption of care? Is it encouraging/enabling overuse/inappropriate use or not?

**Table 3.** Priority Order of Themes Based on Rating Respondent Rankings

Domain	Much more important	More important	Neutral	Less important	Much less important
Clinical appropriateness	39.16%	21.67%	18.33%	11.67%	9.16%
Equity/access	34.17%	20.00%	24.17%	15.00%	6.67%
Clinical parity	25.83%	30.84%	20.83%	17.50%	5.00%
Economics	24.16%	25.83%	16.67%	24.16%	9.16%
Licensing/legal/compliance	20.00%	17.50%	18.33%	20.00%	24.17%
Patient engagement	19.17%	31.67%	25.00%	16.67%	7.50%
Scalability	14.17%	16.66%	29.16%	25.00%	15.00%
Fragmentation	13.33%	18.34%	18.33%	24.17%	25.83%
Team dynamics	5.83%	9.16%	18.33%	25.83%	40.83%
Waste/fraud/abuse	4.16%	8.34%	10.83%	20.00%	56.67%

Based on these preliminary findings, Mayo Clinic Center for Digital Health began impaneling teams of experts to define the key concepts and issues within each of the themes requiring investigation. The goal of these discussions and collaborations is to provide direction for designing the necessary studies to capture the much-needed data as we start implementing these tools and technologies in clinical practice.

## Conclusions

The need for more research to assess the value of virtual care and the lack of standardized payment models and clear guidelines across different payers present significant challenges for providers delivering virtual care. Establishing clear guidelines regarding virtual care payment, standardizing the coverage of virtual care services, and easing the pathways for state-based licensure will help ensure the rapid adoption and success of virtual care.

Payment parity can help ensure that health care providers are compensated fairly for the services rendered to patients and that additional costs of new technology and investment in training and infrastructure can be sufficiently offset. Coverage and payment parity is essential to deliver safe, effective, patient-centered, timely, and efficient virtual care without widening health disparities based on age, insurance, disability, geography, income, race, or ethnicity.

## Abbreviations Used

APM: Alternative Payment Models
CAA: Consolidated Appropriations Act
CMS: Centers for Medicare and Medicaid Services
IMLC: Interstate Medical Licensure Compact
PHE: public health emergency
